# Impact of an online course on enhancing the diagnosis of Chagas disease in Latin America

**DOI:** 10.47487/apcyccv.v5i1.341

**Published:** 2024-03-19

**Authors:** Sebastián García-Zamora, Ricardo López-Santi, Álvaro Sosa-Liprandi, Carina A. Hardy, Andrés F. Miranda-Arboleda, Luis E. Echeverría, José Mauricio Arce, William Uribe, Ezequiel José Zaidel, Luisa Fernanda Aguilera Mora, Darío Di-Toro, Adrián Baranchuk

**Affiliations:** 1 Servicio de Cardiología, Sanatorio Delta, Rosario, Argentina. Servicio de Cardiología Sanatorio Delta Rosario Argentina; 2 Servicio de Cardiología, Hospital Italiano de La Plata, Buenos Aires, Argentina. Servicio de Cardiología Hospital Italiano de La Plata Buenos Aires Argentina; 3 Servicio de Cardiología, Sanatorio Güemes, Buenos Aires, Argentina. Servicio de Cardiología Sanatorio Güemes Buenos Aires Argentina; 4 Servicio de Electrofisiología, Instituto do Coração (Incor), Facultad de Medicina de San Pablo, Brazil. Servicio de Electrofisiología Instituto do Coração (Incor) Facultad de Medicina San Pablo Brazil; 5 Servicio de Arritmias, Brigham and Women’s Hospital, Harvard Medical School, Boston, United States. Servicio de Arritmias Brigham and Women’s Hospital Harvard Medical School Boston United States; 6 Clínica de insuficiencia cardíaca y trasplante, Fundación Cardiovascular de Colombia, Floridablanca, Colombia. Clínica de insuficiencia cardíaca y trasplante Fundación Cardiovascular de Colombia Floridablanca Colombia; 7 Servicio de Arritmias, Instituto Nacional de Tórax, La Paz, Bolivia. Servicio de Arritmias Instituto Nacional de Tórax La Paz Bolivia; 8 Sociedad Inter Americana de Cardiología, Medellín, Colombia. Sociedad Inter Americana de Cardiología Medellín Colombia; 9 Departamento de Farmacología, Facultad de Medicina, Universidad de Buenos Aires, Buenos Aires, Argentina. Universidad de Buenos Aires Departamento de Farmacología Facultad de Medicina Universidad de Buenos Aires Buenos Aires Argentina; 10 Clínica de Insuficiencia Cardiaca, Instituto Cardiovascular de Mínima Invasión, Jalisco, Mexico. Clínica de Insuficiencia Cardiaca Instituto Cardiovascular de Mínima Invasión Jalisco Mexico; 11 Hospital General de Agudos Dr. Cosme Argerich, Buenos Aires, Argentina. Hospital General de Agudos Dr. Cosme Argerich Buenos Aires Argentina; 12 División de Cardiología, Universidad de Queen, Kingston, Ontario, Canada. División de Cardiología Universidad de Queen Kingston, Ontario Canada

**Keywords:** Chagas disease, Electrocardiogram, Medical education, Diagnosis

## Abstract

**Objective.:**

Chagas disease poses a public health problem in Latin America, and the electrocardiogram is a crucial tool in the diagnosis and monitoring of this pathology. In this context, the aim of this study was to quantify the change in the ability to detect electrocardiographic patterns among healthcare professionals after completing a virtual course.

**Materials and Methods.:**

An asynchronous virtual course with seven pre-recorded classes was conducted. Participants answered the same questionnaire at the beginning and end of the training. Based on these responses, pre and post-test results for each participant were compared.

**Results.:**

The study included 1656 participants from 21 countries; 87.9% were physicians, 5.2% nurses, 4.1% technicians, and 2.8% medical students. Initially, 3.1% answered at least 50% of the pre-test questions correctly, a proportion that increased to 50.4% after the course (p=0.001). Regardless of their baseline characteristics, 82.1% of course attendees improved their answers after completing the course.

**Conclusions.:**

The implementation of an asynchronous online course on electrocardiography in Chagas disease enhanced the skills of both medical and non-medical personnel to recognize this condition.

## Introduction

Chagas disease is highly prevalent in Latin America, mainly associated with poverty and adverse socioeconomic conditions ^1^. However, recent migratory trends have led to the global dissemination of this disease, generating increasing interest in high-income regions and countries ^1^^-^^4^. t is estimated that approximately 1 out of every 3 individuals infected with *Trypanosoma cruzi* will develop cardiac involvement over their lifetime, with a latency period of several decades from the initial infection to the disease ^2^^,^^5^^,^^6^. 

The electrocardiogram (ECG) is a valuable tool in the evaluation of individuals at risk of Chagas disease due to its low cost and wide availability. Therefore, it is recommended to perform an annual ECG in individuals with positive serology and without heart disease ^3^^,^^7^^,^^8^, since electrocardiographic abnormalities are related to complications and worse prognosis in this condition ^6^^,^^9^^-^^11^.

Despite being a public health issue in Latin America, the care for Chagas disease has been neglected for decades ^1^. To address this gap, the Inter-American Society of Cardiology (SIAC) implemented a virtual course in Spanish in 2023 with the aim of raising awareness about the disease, encouraging early suspicion and diagnosis, and improving skills in electrocardiographic evaluation among different healthcare team. In this context, the aim of the present study was to quantify the change in the ability to detect electrocardiographic patterns by healthcare professionals after completing a virtual course.

## Materials y methods

### Study design and population

To assess the impact of the course on participants’ knowledge, a non-controlled before-and-after study was conducted using two multiple-choice exams. Both medical and non-medical personnel from Latin America were invited to participate in the course, with no age or profession restrictions. Upon registration for the course, participants were asked to declare their area of work or training. Additionally, medical personnel were asked to specify their specialty, if applicable. Due to the nature of the program, non-probabilistic convenience sampling was used.

### Intervention

A virtual and asynchronous course was developed, with free and open access, on an educational platform dedicated specifically for this purpose (https://www.siacardio.com/academia/ecgchagas/). The main objective of the course was to increase knowledge about ECG for the early detection of Chagas cardiomyopathy among various healthcare team members. To achieve this, seven theoretical classes were created that covered general aspects of the disease, its incidence, prevalence, the importance of early diagnosis, as well as the treatment and care of people living with this disease. Five of the seven modules specifically focused on the different manifestations and alterations of the ECG in this condition. In addition, a virtual library with supplementary materials was made available to the students, expanding on all aspects covered in the course.

The course began in May 2023 and continued until December of the same year. All participants had unrestricted access to the complete course material throughout the period, without any limit on class viewing. 

Each enrolled participant answered a ten-question questionnaire, with four possible options and only one correct answer. At the end of the seven modules of the course, the students had to answer the same exam as the beginning, but with the questions and options presented randomly to prevent mechanical answering.

### Study variables

The dependent variable was whether or not the participant correctly answered 50% or more of the post-test exam questions, and the independent variables were gender, age, the role of the participants in the healthcare system, and, among the doctors, their specialties.

### Statistical analysis

An exploratory descriptive analysis was conducted where the variables were analyzed as mean and standard deviation, assessing normality with graphical tools (histograms and normal probability plots) and the Shapiro-Wilk test. Categorical variables were expressed in absolute values and percentages.

For the comparison of pre- and post-test responses, McNemar’s test was used for paired samples. The internal consistency of the test questions was analysed with Cronbach’s alpha coefficient.

Finally, bivariate regression analysis explored associations between baseline characteristics and knowledge acquisition through the course. For this purpose, logistic regression analysis was carried out between the dependent and independent variables.

Statistical significance was established as a value of p<0.05 in two-tailed tests for all tests performed. Analyses were performed using Stata version 18.0 (Stata Corp., College Station, TX, USA).

### Ethical aspects

The study complied with the Declaration of Helsinki and international ethical guidelines for biomedical research. Confidentiality and data protection were guaranteed, and only the principal investigator had access to personal information. In contrast, all data processing and analysis was conducted anonymously, ensuring confidentiality and protecting the identity of each participant. The project was approved by the SIAC Executive Committee.

## Results

The study included 1,656 participants from 21 countries (Table 1), with an average age of 36.2 ± 10.9 years; 60.3% were men. Of the participants, 87.9% were physicians; among these, 37.1% were cardiologists, 17.4% were internal medicine specialists, 25.0% were family doctors, 6.4% reported having no specialty, 1.7% were pediatricians (including pediatric cardiologists), and the remaining 12.4% had other specialties. Among the 12.1% of non-physician participants, 5.2% were nurses, 4.1% were cardiology practice technicians, and 2.8% were medical students.

**Table 1:** Country of residence of study participants

Country	n	Percentage
Argentina	255	15.4
Colombia	274	16.5
Mexico	202	12.2
Bolivia	160	9.7
Venezuela	230	13.9
Ecuador	102	6.2
Peru	96	5.8
Paraguay	35	2.1
Uruguay	21	1.25
Dominican Republic	14	0.8
Chile	15	0.9
El Salvador	23	1.4
Panama	14	0.8
Costa Rica	20	1.2
Cuba	21	1.25
Guatemala	89	5.4
Honduras	20	1.2
Nicaragua	21	1.3
Spain	23	1.4
Brazil	8	0.5
United States	3	0.2
Did not declare a country	10	0.6

The average rate of correct answers among participants was 23.0% ± 11.7% in the pre-test, with no differences between medical and non-medical personnel (22.9% *vs.* 23.0%, respectively, p=0.910). The percentage of correct answers on the post-test was 55.2% ± 23.4%, with a similar response rate between medical and non-medical participants (p=0.988). No significant differences were observed between medical and non-medical personnel when analyzing each individual response of the pre-test (Table 2).

**Table 2:** Proportion of correct answers of medical and non-medical participants in the pre-test exam

	Non-medical personnel (%)	Medical personnel (%)	p-value
Question 1	26.9	24.9	0.543
Question 2	25.4	24.5	0.796
Question 3	21.9	25.4	0.286
Question 4	21.4	19.9	0.611
Question 5	38.8	37.1	0.642
Question 6	24.4	24.9	0.877
Question 7	22.4	21.1	0.659
Question 8	24.9	25.2	0.932
Question 9	0.5	1.5	0.348
Question 10	23.9	25.0	0.727

The p-values correspond to the McNemar’s test result.

**Figure 1:**
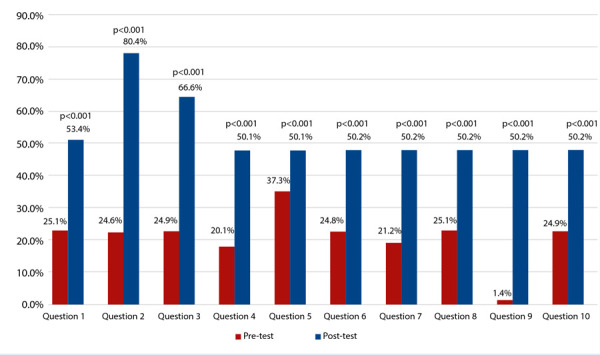
Percentage of correct answers in each question of the exam before (pretest) and after the course (posttest). Los valores de p corresponden al resultado de la prueba de McNemar para la comparación de cada par de preguntas.

3.1% of the course participants correctly answered at least 50% of the pre-test exam questions, a proportion that increased to 50.4% after the course (p=0.001). The increase in the proportion of correct answers occurred across all questions of the questionnaire (p<0.001; Figure 1). 82.1% of the participants answered one or more questions correctly in the post-test evaluation compared to their performance before the course. On the other hand, 9.9% of the participants had the same number of correct answers after the course; finally, 8.0% of the course attendees performed worse in the post-test exam compared to the pre-test evaluation.

The p-values correspond to the result of the McNemar test for comparing each pair of questions.

No differences were found in the baseline characteristics of the participants and the improvement in answers in the post-test exam (Table 3). Because of this, the creation of a multivariable model was not performed.

**Table 3:** Baseline characteristics of participants, and improvement in post-test responses

Variable	Odds ratio	95% CI	p-value
Male gender	0.99	0.81 - 1.20	0.903
Age	1.00	0.99 - 1.01	0.837
Specialty (compared to non-medical personnel) Cardiology Internal medicine, family medicine, and emergency medicine Pediatrics Other specialties No specialty	1.01 1.09 1.12 1.06 1.10	0.72 - 1.38 0.80 - 1.50 0.49 - 2.57 0.69 - 1.64 0.67 - 1.80	0.999 0.587 0.796 0.790 0.707

95% CI: 95% confidence interval.

Finally, the internal consistency of the questionnaire items was assessed using Cronbach’s alpha test, yielding a Cronbach’s alpha value of 0.60, suggestive of good consistency among the questionnaire items.

## Discussion

The main findings of the present study are: i) an asynchronous online e-learning course enhancing the abilities to detect various characteristics and electrocardiographic patterns in Chagas disease; and ii) the positive impact of the course was similar among medical and non-medical personnel, as well as among professionals from different specialties.

More than a decade ago, it was pointed out that the rapid advancement of scientific knowledge in Medicine poses significant challenges for professionals seeking to keep up-to-date ^12^. Although precise data do not exist, it is estimated that, in the 1950s, medical knowledge would double in 50 years, whereas by 2010, this duration had been reduced to 3.5 years ^12^. Currently, it is believed that the timeframe for the duplication of available biomedical knowledge has been shortened to less than a year. This presents a substantial challenge for healthcare professionals across all fields. Technological advances have enabled the implementation of new educational tools, such as augmented or virtual reality, distance learning via interactive platforms, the use of social networks, and, more recently, the integration of artificial intelligence into the teaching-learning process ^13^^-^^15^.

Regarding Chagas disease, multimodality imaging is a crucial tool for comprehensive management of individuals with advanced-stage cardiopathy ^5^. However, the ECG remains fundamental for the diagnosis, prognosis and monitoring of this disease ^1^^-^^3^^,^^6^^-^^10^. Furthermore, its simple and rapid acquisition allows tracings to be obtained by non-medical personnel trained in this task, making it the ideal method for screening a large number of individuals , especially those with difficulties accessing complex healthcare systems, even in suburban and rural areas ^1^^-^^3^. For this reason, it was decided to include non-medical personnel in the program.

Similarly to the present study, other surveys conducted in the region have shown that there are topics inadequately addressed in the training of physicians across different specialties ^16^^-^^18^. However, this work goes further by not just identifying a problem but by presenting an innovative alternative to cope with it. In our study, a brief self-administered educational program for participants had the capacity to enhance participants’ knowledge and ECG interpretation skills in Chagas disease.

The “EDUCATE” clinical trial protocol was recently published, which will explore different approaches to improve learning and interpretation of ECG in various clinical settings ^19^. Unlike this trial, and with its methodological limitations, our study focused on a single pathology, emphasizing the integration of clinical aspects with the varied manifestations of ECG in Chagas disease. It is likely that both strategies will complement each other to provide information on the usefulness of e-learning of electrocardiography applied in different clinical scenarios.

Our study has some limitations that should be considered when interpreting the results. Firstly, the voluntary participation of course attendees could have led to a selection bias towards individuals with greater motivation to acquire knowledge in this area. However, because knowledge is an active process, it is impossible to separate student motivation from the results of a teaching technique. Secondly, our study does not have the ability to assess the persistence of acquired knowledge in the long term. Additionally, it should be noted that the lack of a control group could lead to an overestimation of the true effect of the intervention. Thirdly, multiple-choice assessments are limited methods for assessing students’ critical thinking, reflective capacity, or expression of complex ideas. However, they are efficient tools for evaluating general aspects in a large number of individuals, as in the case of our study. Moreover, in the context of an intervention, these types of assessments allow for an objective assessment of the change in correctly identified responses. Despite the aforementioned limitations and to the authors’ knowledge, this is one of the first educational intervention studies in the region to evaluate the impact of virtual training on improving electrocardiogram interpretation in Chagas disease.

In conclusion, the implementation of an asynchronous, self-administered virtual education program on the value of the electrocardiogram in Chagas disease has allowed for the improvement of healthcare personnel’s skills, both medical and non-medical, in recognizing electrocardiographic abnormalities associated with Chagas cardiomyopathy. Early detection of this pathology is undoubtedly a crucial step towards ceasing it from being a neglected disease. Further studies are necessary to precisely define the role of such interventions in acquiring solid and enduring knowledge, which can be applied in everyday practice across different clinical settings.
